# Lung Cancer in Never-Smokers: A Multicenter Case-Control Study in North China

**DOI:** 10.3389/fonc.2019.01354

**Published:** 2019-12-10

**Authors:** Di Liang, Jingxi Wang, Daojuan Li, Jin Shi, Jin Jing, Baoen Shan, Yutong He

**Affiliations:** Cancer Institute in Hebei Province, The Fourth Hospital of Hebei Medical University, Shijiazhuang, China

**Keywords:** lung cancer, never-smokers, multicenter case-control study, risk factors, protective factors

## Abstract

This study aimed at estimating the effects of epidemiological risk factors for lung cancer in never-smokers. A multicenter and matched case-control study was conducted in the cities of Shijiazhuang, Xingtai, Qinhuangdao, Baoding, and Chengde in North China. It comprised 1,086 cases and 2,172 healthy subjects as controls, all of whom had smoked fewer than 100 cigarettes in their lifetimes. Patients were newly diagnosed with lung cancer between January 2015 and December 2017. Each patient was matched to two control participants for sex and age (±5 years). Both univariate analysis and multivariate conditional logistic regression models were used to estimate the odds ratio (OR) and 95% confidence interval (95% CI). Subsequently, data were stratified by participant sex and different air quality conditions for analysis. Type of job, exposure to environmental tobacco smoke in the workplace or at home, above-average exposure to cooking oil fumes, depression, poor sleep quality, occupational exposure, cardiovascular diseases, and family history of cancer were revealed as significant risk factors for lung cancer in never-smokers. However, higher educational level, frequent use of a PM_2.5_ mask, cooking using clean fuels, and consumption of dietary supplements and tea reduced the risk of lung cancer. Risk factors varied between males and females. In areas with air pollution, the number of risk factors was greater than elsewhere, and the magnitudes of their effects were different. Hence, focusing on these risk factors is important for the prevention and control of lung cancer in never-smokers.

## Introduction

Lung cancer accounts for 11.6% of all newly diagnosed cancer cases and 18.4% of cancer-related deaths worldwide (1). In China, lung cancer is the leading cause of cancer incidence and mortality (2). Several prospective and retrospective studies have reported that smoking to be the major and most well-established risk factor for lung cancer. However, ~15% of males and 53% of females with lung cancer have no history of smoking (3). Approximately 10–20% of lung cancer patients in Europe and the United States are never-smokers, and the proportion of never-smokers among lung cancer patients in Asia is 40–50% (4). In China, 44.9% of cases of lung cancers in males and 86.1% of cases of lung cancers in females are attributed to risk factors other than smoking (5, 6). The proportion of never-smokers in lung cancer patients increased from 31% in 1999–2002 to 48% in 2008–2011 (7). The clinicopathological and molecular mechanisms underlying lung cancer in never-smokers are different from those in smokers. Moreover, mortality from lung cancer in never-smokers was the seventh leading cause of cancer-related death. Furthermore, the incidence rate of lung cancer in never-smokers has been increasing in the last 30 years (8).

The incidence rate of lung cancer is related to the quantity of cigarettes consumed by smokers. Lung cancer incidence among Chinese women is no different from that in Western European countries, despite substantial differences in smoking prevalence (1). Thus, risk factors other than smoking, including genetic susceptibility, poor diet, and occupational exposure, have received considerable attention in recent years (9). However, some studies of lung cancer in never-smokers have only focused on females, and research on lung cancer risk in never-smokers in different air quality areas is limited. Hebei Province, which is in North China, has poor air quality and persistently experiences fog and haze (10, 11). Therefore, a multicenter matched case-control study was conducted to estimate the effects of epidemiological risk factors for lung cancer in never-smokers.

## Materials and Methods

### Study Design and Participants

Participants and controls were recruited between January 1, 2015, and December 10, 2017, in Shijiazhuang City, Baoding City, Xingtai City, Chengde City, and Qinhuangdao City in North China. The first three have poor air quality, and the others have standard air quality.

Two controls were matched for sex and age (±5 years) with one lung cancer patient. The following patients were included in this study: patients who were never-smokers but were diagnosed with primary lung cancer by pathology; patients who were newly diagnosed with lung cancer for the first time between January 1st, 2015, and December 10th, 2017, and were recruited prior to chemotherapy and radiotherapy; patients who were never-smokers (in this study, a never-smoker was defined as someone who had never smoked or smoked fewer than 100 cigarettes in his/her lifetime or someone who stopped smoking longer than 15 years before recruitment) (12); patients residing in local areas (Shijiazhuang City, Baoding City, Xingtai City, Chengde City, and Qinhuangdao City); and patients with no other cancers. Subjects with the following characteristics were included in the control group in this study: never-smokers; participants who were matched for sex and age (±5 years) with cases; participants in the control group with no familial relationship with the matched case; participants with no cancer history; participants with no diseases that could influence lung function. All cases and controls were Han Chinese. There were a total of 1,086 cases (Shijiazhuang: 250 cases from the Fourth Hospital of Hebei Medical University; Baoding: 250 cases collected by the centers for disease control of Baoding City; Xingtai: 245 cases from the People's Hospital of Xingtai city; Chengde: 250 cases from the Third Hospital of Chengde city; Qinhuangdao: 91 cases from the Fourth Hospital of Qinhuangdao city) and 2,172 controls (Shijiazhuang: 500 controls; Baoding: 500 controls; Xingtai: 490 controls; Chengde: 500 controls; Qinhuangdao: 182 controls). This study was approved by the Ethics Committee of the Fourth Hospital of Hebei Medical University.

### Data Collection and Measurements

As part of the recruitment process, participants who enrolled in this study provided written informed consent for inclusion. However, if the participants were under the age of 18 years, both the participants and their parents or guardians provided written informed consent. Subsequently, a trained investigator distributed a structured questionnaire. Participants' basic information (including age, sex, educational level, and occupation), exposure to environmental tobacco smoke (ETS), condition of living quarters, cooking habits, physical exercise habits, stress level, eating habits, alcohol consumption, occupational exposure, history of chronic diseases, and family history of cancer were collected. On-site training and ongoing monitoring were conducted to ensure standardized and uniform data collection between different areas. The database was established by the Hebei Provincial Cancer Institute with double-entry and high-quality control to ensure consistency.

ETS was identified in participants living with a smoker on a regular basis in the workplace or at home (13). The variable “more than 5 kg weight lost” was based on weight loss over the past year, and people who had taken steps to lose weight were not included. For cooking fires, low fire was defined as burning the inner flame of a stove, and high fire was defined as burning the inner and outer flame. Data on exposure to cooking oil fumes, type of cooking fuel, cooking habits, physical exercise habits, and eating habits were continuously and frequently obtained for at least 6 months. Emotional status was defined by several questions that are part of the Eysenck Personality Questionnaire-RSC. High stress was defined as participants feeling tense or anxious for more than 6 months in the previous 3 years. Participants with good mental and social activity the next day and having had more than 6 h of sleep were reported as having experienced good quality sleeping conditions for the previous 1 year. A family history of cancer was defined as participants having first-degree (parents, daughters, sons, brothers, and sisters) or second-degree (uncles, aunts, and grandparents) relatives with cancer. Consumption of dietary supplements was defined as participants' regular intake of vitamins, minerals, herbs, botanicals, or other types of dietary supplements for the previous 30 days (14). Participants consuming alcohol were defined as those imbibing 50 ml of alcohol or 750 ml of beer per day for at least 1 year.

### Statistical Analysis

Demographic variables and risk factors were compared between cases and matched controls using a univariate conditional logistic regression model. Categorical data were presented as numbers and percentages. Multivariate conditional logistic regression models were applied to estimate the odds ratio (OR) and 95% confidence interval (CI) for risk factors with *P* < 0.10 in univariate analysis. All data analyses were performed using Statistical Package for the Social Sciences (SPSS) (version 20, SPSS Inc., Chicago, IL, USA) and Statistical Analysis System (SAS) (version 9.3, SAS Institute Inc., Cary, NC, USA) software. *P* ≤ 0.05, established on two-sided probabilities, was considered statistically significant.

## Results

### Characteristics of the Participants

In this study, a total of 3,258 never-smokers, comprising 1,086 cases with lung cancer and 2,172 healthy controls, were included in the analysis. Females comprised 63.54% (690 cases for the case group, and 1,380 cases for the control group) and males 36.46% (396 cases for the case group, and 792 cases for the control group) of total participants. Participants who were never-smokers with a higher educational level (college or above) had a lower probability of developing lung cancer (OR, 0.43; 95% CI, 0.34–0.54) compared to participants with a lower educational level (senior middle school or below), who were considered the reference group. Compared with other kinds of jobs, farmers or workers who were never-smokers had a higher probability of developing lung cancer (OR, 1.93; 95% CI, 1.64–2.28). Participants who had lost more than 5 kg weight in the previous year had a 51% increased risk of lung cancer (OR, 1.51; 95% CI, 1.23–1.87) (Table 1).

**Table 1:** General characteristics of lung cancer case group and control group in never-smokers.

**Factors**	**Case *N* (%)**	**Control *N* (%)**	***P*-value**	**OR**	**95% CI**
**Education**			<0.001		
Senior middle school or below	965 (29.62)	1,731 (53.13)		1.00	Reference
College or above	121 (3.71)	441 (13.54)		0.43	0.34, 0.54
**Job**			<0.001		
Other	426 (13.08)	1,141 (35.02)		1.00	Reference
Farmer/Worker	660 (20.26)	1,031 (31.65)		1.93	1.64, 2.28
**More than 5 kg weight lost**			<0.001		
No	912 (27.99)	1,931 (59.27)		1.00	Reference
Yes	174 (5.34)	241 (7.40)		1.51	1.23, 1.87

*OR, Odds ratio; 95% CI, 95% confidence interval*.

### Exposure to Environmental Tobacco Smoke and Smog

A significant association was observed between lung cancer and ETS in the workplace or at home. A 2.23-fold increased risk (OR, 2.23; 95% CI, 1.84–2.71) and a 2.33-fold increased risk (OR, 2.33; 95% CI, 1.99–2.72) of lung cancer were observed for never-smokers who were exposed to smoke in the workplace and at home. Using a fine particulate matter (PM_2.5_) mask (OR, 0.26; 95% CI, 0.17–0.40) and cooking using clean fuels (OR, 0.56; 95% CI, 0.47–0.66) were revealed as protective factors. Compared to participants who cooked 0–1 time per day, increased risk of lung cancer was observed in participants who cooked 2–3 times per day (OR, 1.36; 95% CI, 1.08–1.71). Compared to smokeless cooking, both normal exposure (OR_normal_, 2.49; 95% CI, 1.91–3.25) and above-average exposure (OR_more_, 3.08; 95% CI, 2.31–4.11) to cooking oil fumes were significantly associated with lung cancer (Table 2).

**Table 2:** Estimated risks of lung cancer associated with ETS, smog, and cooking habits in never-smokers.

**Factors**	**Case *N* (%)**	**Control *N* (%)**	***P*-value**	**OR**	**95% CI**
**ETS exposure in the workplace**			<0.001		
No	706 (21.67)	1,669 (51.23)		1.00	Reference
Yes	380 (11.66)	503 (15.44)		2.23	1.84, 2.71
**ETS exposure at home**			<0.001		
No	453 (13.90)	1,335 (40.98)		1.00	Reference
Yes	633 (19.46)	837 (25.69)		2.33	1.99, 2.72
**Frequent use of a PM**_**2.5**_ **mask**			<0.001		
No	1,060 (32.54)	1,995 (61.23)		1.00	Reference
Yes	27 (0.83)	176 (5.40)		0.26	0.17, 0.40
**Decoration in your home**			0.003		
No	714 (21.92)	1,535 (47.11)		1.00	Reference
Yes	372 (11.42)	637 (19.55)		1.29	1.09,1.52
**Clean fuels for cooking**			<0.001		
No	386 (11.85)	543 (16.67)		1.00	Reference
Yes	700 (21.49)	16,289 (50.00)		0.56	0.47, 0.66
**Using coal for cooking**			<0.001		
No	924 (28.83)	1,916 (59.78)		1.00	Reference
Yes	160 (4.99)	205 (6.40)		1.90	1.48, 2.45
**Cooking fire**			<0.001		
Low fire	60 (1.84)	141 (4.33)		1.00	Reference
Moderate fire	828 (25.41)	1,467 (45.03)	0.052	1.38	0.99, 1.91
High fire	198 (6.08)	564 (17.31)	0.162	0.78	0.54, 1.11
**Cooking frequencies**			0.008		
0–1 time/day	162 (4.97)	395 (12.12)		1.00	Reference
2–3 times/day	924 (28.36)	1,777 (54.54)		1.36	1.08, 1.71
**Cooking-oil fumes**			<0.001		
Smokeless	98 (3.01)	399 (12.25)		1.00	Reference
Normal	720 (22.10)	1,370 (42.05)	<0.001	2.49	1.91, 3.25
Above-average	268 (8.23)	403 (12.37)	<0.001	3.08	2.31, 4.11

### Disease History, Lifestyle Habits, Family History of Cancer, and Occupational Exposure

The ORs of cardiovascular diseases, diabetes, and diseases of the digestive system, considered as chronic diseases, were 1.77 (95% CI, 1.47–2.13), 1.33 (95% CI, 0.98–1.79), and 1.23 (95% CI, 0.92–1.63), respectively. Depression (OR, 1.65; 95% CI, 1.39–1.94) and high stress (OR, 1.49; 95% CI, 1.18–1.90) were revealed as risk factors for lung cancer. Quality of sleep was associated with lung cancer, with poor sleep quality being harmful to participants' health (OR, 1.29; 95% CI, 1.10–1.52). A family history of cancer was significantly associated with increased risk (OR, 1.57; 95% CI, 1.26–1.96) of lung cancer. However, physical exercise habits and lung cancer showed no significant association (Table 3).

**Table 3:** Estimated risks of lung cancer associated with emotion, lifestyle, occupational exposures, history of disease, and family history of cancer in never-smokers.

**Factors**	**Case *N* (%)**	**Control *N* (%)**	***P*-value**	**OR**	**95% CI**
**Emotion**			<0.001		
Happiness	485 (14.89)	1,183 (36.31)		1.00	Reference
Depression	601 (18.45)	989 (30.36)		1.65	1.39, 1.94
**High stress**			0.001		
No	943 (28.94)	1,966 (60.34)		1.00	Reference
Yes	143 (4.39)	206 (6.32)		1.49	1.18, 1.90
**Sleeping quality**			0.002		
Good quality	720 (22.10)	1,551 (47.61)		1.00	Reference
Poor quality	366 (11.23)	621 (19.06)		1.29	1.10, 1.52
**Physical exercise**			0.542		
No	606 (18.60)	1,187 (36.43)		1.00	Reference
Often	480 (14.73)	985 (30.23)		0.95	0.82, 1.10
**Occupational exposure**			<0.001		
No	890 (27.32)	1,959 (60.13)		1.00	Reference
Yes	196 (6.02)	213 (6.54)		2.37	1.87, 3.00
**Cardiovascular diseases**			<0.001		
No	784 (24.06)	1,760 (54.02)		1.00	Reference
Yes	302 (9.27)	412 (12.65)		1.77	1.47, 2.13
**Diabetes**			0.072		
No	1,010 (31.00)	2,054 (63.04)		1.00	Reference
Yes	76 (2.33)	118 (3.62)		1.33	0.98, 1.79
**Digestive system diseases**			0.164		
No	1,002 (30.76)	2,032 (62.37)		1.00	Reference
Yes	84 (2.58)	140 (4.30)		1.23	0.92, 1.63
**Family history of cancer**			<0.001		
No	916 (28.12)	1,935 (59.39)		1.00	Reference
Yes	170 (5.22)	237 (7.27)		1.57	1.26, 1.96

### Dietary Factors

Fruit intake 3–5 days per week (OR, 0.79; 95% CI, 0.65–0.95) and 6–7 days per week (OR, 0.34; 95% CI, 0.27–0.42) in never-smokers was considered a protective factor for lung cancer. The frequent consumption of spicy food was not significantly associated with lung cancer. However, there was an increased risk of lung cancer for specific types of pepper (fresh pepper, dry pepper, and pepper oil), while chili sauce and other types of pepper had no significant association with lung cancer. The consumption of high-degree liquor was significantly associated with lung cancer (OR, 1.59; 95% CI, 1.08–2.32), while the consumption of low-degree wine and beer had no significant association. On the other hand, the consumption of dietary supplements (OR, 0.27; 95% CI, 0.16–0.46) and tea (OR, 0.66; 95% CI, 0.54–0.80) reduced the risk of developing lung cancer (Table 4).

**Table 4:** Estimated risks of lung cancer associated with dietary factors in never-smokers.

**Factors**	**Case *N* (%)**	**Control *N* (%)**	***P*-value**	**OR**	**95% CI**
**Vegetables**			<0.001		
≤2 days per week	138 (4.24)	239 (7.34)		1.00	Reference
3–5 days per week	395 (12.12)	614 (18.85)	0.879	1.02	0.77, 1.36
6–7 days per week	553 (16.97)	1,319 (40.48)	0.002	0.64	0.49, 0.85
**Fruits**			<0.001		
≤2 days per week	357 (10.96)	495 (15.19)		1.00	Reference
3–5 days per week	511 (15.68)	860 (26.40)	0.010	0.79	0.65, 0.95
6–7 days per week	218 (6.69)	817 (25.08)	<0.001	0.34	0.27, 0.42
**Spicy food**			0.722		
No	903 (27.72)	1,816 (55.74)		1.00	Reference
Often	183 (5.62)	356 (10.93)		1.04	0.84, 1.28
**Fresh pepper**			<0.001		
No	896 (27.50)	1,939 (59.52)		1.00	Reference
Often	190 (5.83)	233 (7.15)		1.84	1.48, 2.28
**Dry pepper**			0.003		
No	939 (28.82)	1,952 (59.91)		1.00	Reference
Often	147 (4.51)	220 (6.75)		1.44	1.14, 1.82
**Pepper oil**			0.001		
No	1,006 (30.88)	2,072 (63.60)		1.00	Reference
Often	80 (2.46)	100 (3.07)		1.68	1.23, 2.29
**Dietary supplements**			<0.001		
No	1,070 (32.84)	2,065 (63.38)		1.00	Reference
Yes	16 (0.49)	107 (3.28)		0.27	0.16, 0.46
**Alcohol consumption**			1.000		
No	881 (27.04)	1,763 (54.11)		1.00	Reference
Yes	205 (6.29)	409 (12.55)		1.00	0.80, 1.24
**Low-degree liquor**			0.066		
No	978 (30.02)	1,914 (58.75)		1.00	Reference
Yes	108 (3.31)	258 (7.92)		0.78	0.61, 1.02
**High-degree liquor**			0.017		
No	1,030 (31.61)	2,097 (64.36)		1.00	Reference
Yes	56 (1.72)	75 (2.30)		1.59	1.08, 2.32
**Beer**			1.000		
No	1,049 (32.20)	2,098 (64.40)		1.00	Reference
Yes	37 (1.14)	74 (2.27)		1.00	0.66, 1.53
**Tea consumption**			<0.001		
<2 days per week	843 (25.87)	1,551 (47.61)		1.00	Reference
≥2 days per week	243 (7.46)	621 (19.06)		0.66	0.54, 0.80

### Multivariate Analysis of Risk Factors

Table 5 shows the significant associations between risk factors and lung cancer in never-smokers. Higher educational level, frequent use of a PM_2.5_ mask, regular fruit intake, cooking using clean fuels, and consumption of dietary supplements and tea were revealed as protective factors for lung cancer in never-smokers. Compared with other types of jobs, farmers and workers had a higher probability of developing lung cancer (OR, 1.27; 95% CI, 1.03–1.57). ETS exposure in the workplace (OR, 1.93; 95% CI, 1.53–2.42) or at home (OR, 1.93; 95% CI, 1.62–2.31) was associated with lung cancer. Normal exposure (OR, 1.59; 95% CI, 1.18–2.13) and above-average exposure (OR, 1.70; 95% CI, 1.22–2.38) to cooking oil fumes were significantly associated with lung cancer. Occupational exposure (OR, 1.61; 95% CI, 1.23–2.10) increased the risk of lung cancer. Poor sleep quality, depression, cardiovascular diseases, and family history of cancer in never-smokers were shown to be risk factors for lung cancer (Table 5).

**Table 5:** Estimated risks of lung cancer associated with multivariate factors in never-smokers.

**Factors**	***P*-value**	**OR**	**95% CI**	
			**Lower**	**Upper**
**Education**	0.007			
Senior middle school or below		1.00	Reference	
College or above		0.67	0.50	0.90
**Job**	0.020			
Other		1.00	Reference	
Farmer/worker		1.27	1.03	1.57
**ETS exposure in the workplace**	<0.001			
No		1.00	Reference	
Yes		1.93	1.53	2.42
**ETS exposure at home**	<0.001			
No		1.00	reference	
Yes		1.93	1.62	2.31
**Frequent use of a PM**_**2.5**_ **mask**	<0.001			
No		1.00	Reference	
Yes		0.30	0.19	0.50
**Cooking-oil fumes**	0.003			
Smokeless		1.00	Reference	
Normal	0.002	1.59	1.18	2.13
Above-average	0.002	1.70	1.22	2.38
**Clean fuels for cooking**	0.020			
No		1.00	Reference	
Yes		0.78	0.63	0.96
**Emotion**	0.007			
Happiness		1.00	Reference	
Depression		1.31	1.07	1.59
**Sleeping quality**	<0.001			
Good quality		1.00	Reference	
Poor quality		1.48	1.22	1.79
**Occupational exposure**	0.001			
No		1.00	reference	
Yes		1.61	1.23	2.10
**Cardiovascular diseases**	<0.001			
No		1.00	Reference	
Yes		1.53	1.24	1.89
**Family history of cancer**	<0.001			
No		1.00	Reference	
Yes		1.92	1.47	2.51
**Fruits**	<0.001			
≤2 days per week		1.00	Reference	
3–5 days per week	0.070	0.82	0.66	1.01
6–7 days per week	<0.001	0.45	0.36	0.57
**Dietary supplements**	<0.001			
No		1.00	Reference	
Yes		0.29	0.15	0.54
**Tea consumption**	0.002			
<2 days per week		1.00	Reference	
≥2 days per week		0.70	0.56	0.88

**Figure 1:**
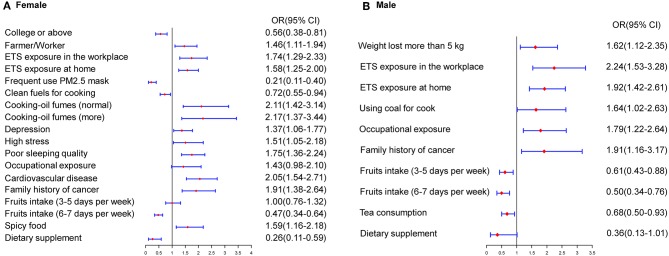
Estimated risks of lung cancer in never-smokers by gender. **(A)** Multivariate risk factors model in females. **(B)** Multivariate risk factors model in males.

Participants were categorized by sex for analysis. According to the multivariate model, the significant predictive factors of lung cancer in females were similar to those in the overall model. In females, a higher educational level, consumption of dietary supplements, frequent use of a mask, cooking using clean fuels, and regular fruit intake were identified as protective factors, while ETS exposure in the workplace (OR, 1.74; 95% CI, 1.29–2.33) or at home (OR, 1.58; 95% CI, 1.25–2.00) and family history of cancer (OR, 1.91; 95% CI, 1.38–2.64) were identified as risk factors. Furthermore, above-average exposure to cooking oil fumes, specific types of jobs, and occupational exposure significantly increased the risk of lung cancer in never-smokers. Moreover, participants' mental states and habits, including depression, high stress, poor sleep quality, and frequent consumption of spicy food, were revealed as risk factors for lung cancer in never-smokers. In males, consumption of tea (OR, 0.68; 95% CI, 0.50–0.93) and regular fruit intake were significant protective factors. ETS exposure in the workplace or at home were risk factors in males, with ORs of 2.24 and 1.92, respectively. Occupational exposure, above-average exposure to cooking oil fumes, weight loss of more than 5 kg, and family history of cancer were significantly associated with lung cancer (Figure 1).

**Figure 2:**
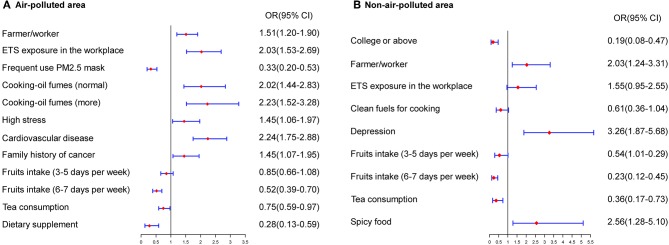
Estimated risks of lung cancer in never-smokers by air quality. **(A)** Multivariate risk factors model in air-polluted areas. **(B)** Multivariate risk factors model in non-air-polluted areas.

The risk factors for lung cancer differed between areas with and without air pollution. The multivariate model for polluted areas included a series of variables as related factors for lung cancer, such as type of job, occupational exposure, ETS exposure in the workplace, high pressure, cooking using clean fuels, frequent use of a PM_2.5_ mask, family history of cancer, cardiovascular diseases, consumption of dietary supplements and tea, and regular fruit intake. In areas without air pollution, variables such as type of job, ETS exposure in the workplace or at home, educational level, family history of cancer, regular fruit intake, consumption of tea, frequent consumption of spicy foods, and depression were significantly associated with lung cancer in never-smokers (Figure 2).

## Discussion

Studies on lung cancer have revealed that risk factors, including genetic susceptibility, poor diet, and occupational exposure, might either be considered as independent risk factors for lung cancer or be associated with smoking-related lung cancer. Risk factors differ between smoking-related and non-smoking-related lung cancer, especially with respect to sex and air quality (15–17). In China, several studies have aimed at confirming the above hypothesis, but corroborating results have been limited. The present study used epidemiological data to assess the risk factors and protective factors significantly associated with lung cancer in never-smokers and carried out stratified analysis by sex and air quality in North China.

According to previous studies, a critical risk factor for lung cancer was smoking, with population-attributable risk proportions of 75.04% and 18.35% in males and females, respectively (6). Tobacco smoking causes deoxyribonucleic acid damage in bronchial epithelial cells, causing dysfunction in the immune system in the lungs. In some Asian regions, smoking rates in females are low, and the incidence rates of female lung cancer are inconsistent with expected incidence rates (18). According to a retrospective analysis of data from 192 countries, ~21,000 deaths by lung cancer could be attributed to secondhand smoking (19). In China, 740 million never-smokers are exposed to secondhand smoke, resulting in ~100,000 deaths (20). In this study, ETS exposure in the workplace (OR, 1.93; 95% CI, 1.53–2.42) or at home (OR, 1.93; 95% CI, 1.62–2.31) had a significant effect on lung cancer. Similarly, a large-scale prospective European study estimated that the proportion of lung cancers in never- and ex-smokers attributable to ETS was between 16 and 24%, mainly due to work-related exposure (21), while a few studies reported lower proportions of ETS exposure in the living place compared to the abovementioned study. Gorlova et al. reported that ETS exposure in the workplace (OR, 3.94; 95% CI, 1.54–10.06) was more significantly associated with lung cancer than ETS exposure at home (OR, 2.02; 95% CI, 1.06–3.85) (13). “Sidestream” smoke, which is defined as the smoke released from the end of a burning cigarette, could directly contribute to 80% of the smoke in secondhand smoking. Because sidestream smoke is generated at a lower temperature than “mainstream” smoke, it can lead to a thicker density of smoke containing at least 17 kinds of carcinogens compared to mainstream smoke (22).

This study found that males and females were differently affected by ETS exposure. The OR of secondhand smoking was higher for males than for females, with ORs of ETS exposure at home of 1.92 and 1.58 for males and females, respectively. In the workplace, the OR for females (1.74) was lower than that for males (OR, 2.24). An International Agency for Research on Cancer (IARC) analysis estimated that the risk of lung cancer was increased by more than 35% in males and 25% in females who were exposed to secondhand smoke compared to non-exposed males and females (23). Approximately 26.51% of cases of never-smokers with undiagnosed lung cancer can be attributed to passive smoking; thus, reducing exposure to secondhand smoke in never-smokers is beneficial with respect to the prevention and treatment of lung cancer (24).

According to some studies, a diet rich in vegetables and fruits, especially cruciferous vegetables, may help prevent lung cancer. However, the results of several studies with detailed information on dietary intake are inconsistent (25, 26). This study revealed that regular fruit intake and consumption of tea and dietary supplements were protective factors for lung cancer in never-smokers. Dose-response relationships for diets rich in fruits were identified in this study. Compared to participants who consumed fruits <2 days per week, the adjusted OR was lower (OR, 0.45; 95% CI, 0.36–0.57) for participants who consumed fruits 6 or 7 days per week. Some case-control studies also revealed that never-smokers who regularly consumed fruits had a decreased risk of developing lung cancer. According to a study by Vieira et al., regular fruit intake in never-smokers had no significant association with lung cancer, with a relative risk of 0.88 (95% CI, 0.68–1.15) (25). The inconsistency between the results of the studies may be attributable to the different study populations and confounding factors of lung cancer, such as passive smoking. Moreover, the target population in this study were Chinese, and we performed multivariate analysis to control for confounding factors. The protective effects of fruits may be due to biologically active compounds, such as sulforaphane, glucosamine, and indole derivatives, which have antioxidant functions. Tea contains several catechins, such as epigallocatechin gallate, epigallocatechin, and epicatechin gallate, which prevent the development of cancer (27). Consumption of tea and regular fruit intake prevent the development of diseases in humans.

Regarding the Chinese population, their preferred cooking methods are one of the risk factors of lung cancer. Compared to smoke-free cooking, the OR of above-average exposure to cooking oil fumes in never-smokers with lung cancer was 1.70 (95% CI, 1.22–2.38) in this study. Studies also revealed that different types of fuels could be considered to result in different risks of lung cancer, and cooking using clean fuels could be a protective factor in lung cancer, with an OR of 0.78 (95% CI, 0.63–0.96). Furthermore, Mu et al. reached similar conclusions: individuals with no separate kitchen had higher risk of lung cancer than those with a separate kitchen. Additionally, the absence of ventilation equipment could increase the risk of lung cancer (OR, 1.78; 95 CI, 1.09–2.90) (28). Cooking fuels generated from unclean fuels and high-temperature cooking have become one of the most serious indoor environmental pollutants, and they were classified as indoor carcinogenic pollutants by the IARC (25, 29). Completely eliminating these harmful cooking habits could reduce the risk of developing lung cancer in never-smokers.

This study revealed that a family history of lung cancer could contribute to the development of lung cancer in never-smokers, increasing risk 1.92-fold (95% CI, 1.47–2.51). The ORs of a family history of lung cancer in male and female never-smokers were 1.91 (95% CI, 1.38–2.64) and 1.91 (95% CI, 1.16–3.17), respectively. Gorlova et al. revealed that the risk of lung cancer in females was significantly higher compared to in males (21). The results of this study were similar to those of Lo et al. (24). Another meta-analysis revealed that a family history of cancer was closely associated with lung cancer in never-smokers, with a 1.5-fold increased risk (30). Hence, individuals who have a family history of cancer should undergo regular screening for lung cancer.

The Global Burden of Diseases, Injuries, and Risk Factors Study 2015 (GBD 2015) reported that exposure to air pollution could increase the incidence and mortality of lung cancer and is the leading cause of the global disease burden (31). Air pollution is a major, insufficiently appreciated cause of non-communicable disease. Some other risk factors for lung cancer in never-smokers might be influenced by air pollution and differed in areas with different air qualities. Particle mass with an aerodynamic diameter < 2.5 μm (PM_2.5_) is the most consistent and robust predictor of mortality (32, 33). Long-term exposure to PM_2.5_ has been associated with an increased risk of developing lung cancer (34). According to data from China's Ministry of Environmental Protection, the cities of Baoding, Shijiazhuang, and Xingtai are among the most heavily polluted cities in China, with PM_2.5_ concentrations of ~60–80 μg/m^3^ per year. The PM_2.5_ concentrations in Chengde and Qinhuangdao were reported to be below 30 μg/m^3^ per year (35). The relative risk of PM_2.5_ and lung cancer mortality in Hebei province was 1.20 (95% CI, 1.10-1.26) for females (35). A study by Gharibvand et al. also reached a similar conclusion, that PM_2.5_ might contribute to lung cancer incidence (HR, 1.43; 95% CI, 1.11–1.84) in never-smokers (36). Exposure in the workplace and air pollution might have synergistic effects. In air polluted areas, the adjusted OR of ETS exposure was higher (OR, 2.03; 95% CI, 1.53–2.69) than that of all areas (OR, 1.93; 95% CI, 1.53–2.42). After air quality conditions were stratified, above-average exposure to cooking oil fumes was significantly more harmful than smokeless exposure (OR, 2.23; 95% CI, 1.52–3.28). Furthermore, household air pollution from the burning of fuels has also been shown to be a major cause of lung cancer in low-income and middle-income countries and, with ambient air pollution, poses a substantial public health challenge (31). Because of the relationship between PM_2.5_ and lung cancer, it could lead to variation in the effect magnitude in the other risk factors. Hence, the data was stratified by air quality so as to provide more information to targeted prevention. In areas with air pollution, individuals should protect themselves from ETS and exposure cooking oil fumes and should wear a PM_2.5_ mask frequently to prevent the occurrence of lung cancer.

This study has the following strengths: a sufficiently large sample size and the use of stratified analysis by sex and air quality conditions. The epidemiological risk factors for lung cancer were exposure to passive smoking, cooking habits, disease history, and family history of cancer. This study has the limitation that smoking status and other variables were self-reported. Hence, never-smokers may have been misclassified, especially participants who were ex-smokers and had stopped smoking <15 years before. The development of lung cancer is a complex process, as it involves several factors, including epidemiological and genetic factors and gene–environment interactions.

## Data Availability Statement

All datasets generated for this study are included in the article/supplementary material.

## Ethics Statement

The studies involving human participants were reviewed and approved by This study was approved by the Ethics Committee of Hebei Medical University Fourth Hospital. Written informed consent to participate in this study was provided by the participants' legal guardian/next of kin.

## Author Contributions

DLia and YH wrote the main text and performed data analysis. YH and BS designed the study. DLi, JS, JJ, and JW collected the data. All authors reviewed the manuscript.

### Conflict of Interest

The authors declare that the research was conducted in the absence of any commercial or financial relationships that could be construed as a potential conflict of interest.
